# Breast Carcinoma Progression and Tumour Vascular Markers Related to Apoptotic Mechanisms

**DOI:** 10.1155/2014/156034

**Published:** 2014-02-18

**Authors:** Miroslava Bilecova-Rabajdova, Peter Urban, Kristina Gregova, Jan Varga, Viera Fialkovicová, Peter Kruzliak, Maria Marekova

**Affiliations:** ^1^Department of Medical and Clinical Biochemistry and LABMED, Faculty of Medicine, P. J. Šafárik University in Košice, Trieda SNP 1, 040 11 Košice, Slovakia; ^2^Department of Histology and Embryology, Faculty of Medicine, P. J. Šafárik University in Košice, Šrobárova 2, 040 11 Košice, Slovakia; ^3^2nd Department of Gynecology and Obstetrics, Faculty of Medicine, P. J. Šafárik University in Košice, Rastislavova 43, 040 11 Košice, Slovakia; ^4^International Clinical Research Center, St. Anne's University Hospital, Pekařská 53, 656 91 Brno, Czech Republic

## Abstract

*Background*. In the last few years, the cancer research had tried to identify and characterize new biochemical and molecular pathways in which the inhibition induces prosurvival mechanisms. Our work describes the expression of two different members of apoptotic regulatory pathway and their relationship with a progression of breast carcinoma. *Materials and Methods*. We compared expression of genes related to apoptosis (*DR6 *and *Gpm6B*) in the blood of patients suffering from stage I of breast cancer in different grades (I–IV), with healthy controls. After isolation of mRNA, transcription of mRNA into the cDNA was performed. The quantification of gene expression changes in *DR6 *and *Gpm6B *was detected by RT-PCR method. Analysis at the protein level was performed by the Western blot.*Results*. In statistical analysis of *Dr6 *mRNA level changes we detected significant increase starting in Grading 1 (G1) and reached maximal level in G3.This expression on mRNA levels was similar to protein levels, which copy rising tendency with maximal value in G3. The results of *Gpm6B *were significantly lower.*Conclusion*. This result showed that antiapoptotic signalling during neovascularization is increased significantly. It would be advisable in the future to study the influence of cytostatic treatment on the expression of genes related to apoptotic pathways and their relationship with progression of breast cancer tumours.

## 1. Introduction

Normal cell development is controlled by a balance between cell proliferation and apoptosis, and there is strong evidence that tumour growth is not only a result of uncontrolled proliferation but also a result of reduced apoptosis. The balance between proliferation and apoptosis is crucial in determining the overall growth or regression of the tumour. Cancer cells overcome cell death or apoptosis due to mutation of tumour suppressor genes and impaired apoptotic machinery executing cell death [1]. Another essential process important for the growth and metastasis of solid tumours is angiogenesis. The balance of endothelial cell (EC) proliferation and apoptosis is a major determinant in tumour angiogenesis, endothelial cell survival, and apoptosis in the tumour vasculature [2]. For malignant growth, solid cancers must stimulate the formation of new blood vessels by producing vascular endothelial growth factor (VEGF-A), which is required for the survival of tumour-associated vessels.

As a result of higher levels of VEGF-A, Seaman et al. [3] pointed out utilization of tumour-endothelial markers during carcinoma differentiation. Endothelial cell of the tumour, population of perivascular cells, and vascular leukocytes (VLC) are the producers of specific tumour vascular markers (TVM) [4]. To assess the level of TVM expression (*DR6* and *Gpm6B*) and explore its potential pro- and antiapoptotic role during tumour development, we examined the expression of *DR6* and *Gpm6B* mRNAs by RT-PCR and Western blot analysis isolated from human peripheral whole blood. These TVM were specifically chosen according to the journal paper of Buckanovich et al. [5], who declared that these markers are suitable for the detection of breast tumours. The overexpression of *DR6* and *Gpm6B* leads to abnormal signalling pathways that contribute to the regulation of apoptotic processes in EC and tumour cells.

Our work describes the expression of two different members of apoptotic regulatory pathway and their relationship with a progression of breast carcinoma. This approach could help with the development of effective antiangiogenic therapies, which require identifying biologically relevant molecular targets associated with increased tumour angiogenesis [6].

The first TVM with antiapoptotic activity whose expression was examined is glycoprotein M6B (*Gpm6B*). This glycoprotein is a transmembrane protein related to the proteolipid protein (PLP) family that also includes M6A and the smaller splice variant DM20 [7]. Just recently, the membrane glycoprotein M6A was demonstrated to interact with the *μ*-opioid receptor, an interaction that facilitates endocytosis and recycling of the receptor [8]. The function of *Gpm6B* is still largely unknown, although it has been speculated that proteolipid proteins, in addition to their role in structuring of the myelin, might also function as housekeeping proteins involved in intracellular trafficking [9]. Fjorback et al. [10] discovered that coexpression of SERT with *Gpm6B* mediates a significant decrease in SERT-mediated 5-HT-uptake. In the presence of *Gpm6B*, the 5-HT uptake was decreased by 50%. Previous studies on 5-HT physiology in mammary gland cells revealed the critical roles of 5-HT in regulating epithelial homeostasis during involution, which is characterized by epithelial tissue regression [11]. One expected effect of elevated 5-HT activity in the normal breast is widespread apoptotic cell death [12].

The second studied TVM with proapoptotic activity was death receptor 6 (*DR6*). This protein belongs to tumour necrosis factor group (TNF), specifically to death receptor group (DRS: death receptors system). *DR6* like other DRS plays role in regulation of cell-specific and inflammatory response of the cell. The primary role is apoptosis induction, but they play role also in cell proliferation, differentiation, and programmed cell death [13]. TNF receptors, in their extracellular part, contain many cysteine residues that are able to bind ligands. After binding ligand to receptor, by the effect of bond and death domain, caspase is activated and apoptosis is initiated. The function of *DR6* in apoptosis activation is not clear, because *DR6* ligand was not detected yet. The activation of apoptosis by *DR6*, TRAIL/apoL receptors, or NF-UB, JNK pathways together with TNFR1and CD95 is clarified. High expression of *DR6* in carcinoma cells that influences antitumour's response by differentiate and proliferative effects on monocytes correlates with high activity of NF-kB [14]. Zeng et al. [15] suggest that *DR6*-induced apoptosis occurs through a new pathway that is different from the classical pathways mainly through interacting with Bax.

## 2. Material and Methods

### 2.1. Experimental Design

A total of 110 examined patients were participating in experiment. The experimental group consists of patients (*n* = 75) with breast carcinoma (ductal invasive carcinoma *n* = 65; average of age *X* = 53, lobular invasive carcinoma *n* = 8; average of age *X* = 57, and nondifferentiated breast carcinoma *n* = 2, average of age *X* = 44). All patients in the experimental group had a tumour with the size of stage I, but individual grade of tumours was different. The control group consists of 35 women (*n* = 35; average of age *X* = 51). Women in the control group measured blood pressure and clinical biochemistry results were evaluated along with the overall health status evaluated in standard preventive examinations. Patients and those in the control group responded to the questionnaire. The control group that consisted of people who feel subjectively healthy and preventive examination by the doctor was negative with regard to the current troubles in their health. Screening on comarkers was investigated in the range of reference values for various tumour markers and just had negative sonographic examination of the reproductive organs. Tumour predisposition we take into account with regard to the incidence of cancer among relatives in previous generations. All women in the control group (*n* = 35) were subjected to standard haematological and clinical biochemical laboratory tests (e.g., blood count, coagulation tests, APTT: activated practical thromboplastin time, bleeding time, Quick test, recalcification time, glycemia, lipid profile, total protein, albumin, bilirubin, AST, ALT, ALP, amylase, uric acid, and qualitative urine analysis), which were done by routine haematological and biochemical analytical procedures using bionalyzer Advia, Sysmex Centaur, in cooperation with the complex diagnostic laboratories LABMED in Košice.

### 2.2. Immunohistochemical Analysis

In all patients from the experimental group (*n* = 75) histopathological and cytological examination was performed, for confirmation or for establishing the diagnosis. In the laboratory CytoLab Ltd. and the Department of Pathology UNLP in Kosice, health care professionals used the tumour tissue and subjected them to excitation and arrangements necessary for the evaluation of histological slides. Stage and individual grades of tumours were confirmed by histological and immunohistological methods. The basic markers for the determination of breast tumour progression, such as estrogenic receptor clone 1D5, progesterone receptor PgR636, clone D07 of apoptotic protein p53, and Ki67 clone MIB1, were examined by the Department of Laboratory Medicine, University Hospital of L. Pasteur in Košice, using peripheral blood of patients.

### 2.3. RT-PCR Analysis

To find evidence of changes in mRNA levels, we decided to use RT-PCR. We performed four analyses for each gene per person in experimental and control groups. Blood has been taken from *vena mediana cubiti* to K_2_EDTA covered test tube. RNA was isolated from peripheral blood diagnostic isolation kit (Qiagen). The reverse transcription from mRNA to cDNA was made using superscript II (Invitrogen). Normalization of the results was performed by housekeeping gene *β*-actin. Amplification of the specific genes *DR6*, *Gpm6B,* and **β*-actin *ran for 30 cycles (94°C 5 min, 94°C 30 s, 58.4°C 30 s, and 72°C 45 s), using appropriate primers (Table 1) in the thermocycler 7500 Real-Time PCR System (Applied Biosystems). Electrophoresis of individual genes' PCR products was performed on agarose gels. Subsequent quantification was done by measuring fluorescent signal intensity using the GBOX visualization system (Syngene). Numerical quantification of changes in expression levels was evaluated using the DataSyngene program.

### 2.4. Western Blot Analysis

The proteins from peripheral blood were isolated by using SQ DNA/RNA/protein kit (Omega) and resolved by sodium dodecyl sulphate-polyacrylamide gel electrophoresis (SDS-PAGE). After western blotting (Trans blot SD semidry transfer cell, Biorad) blots on nitrocellulose membrane were probed with a rabbit monoclonal antibody against *Gpm6B* (Sigma, dilution 1 : 100) at room temperature for 12 hours and rabbit monoclonal antibody against *DR6* (Sigma) at room temperature for 5 hours in dilution 1 : 1000. For normalization of our data we used *β*-actin antibody (Santa Cruz Biotechnology). After being washed by 0.05% phosphate buffered saline (PBS)-Tween, the membranes were incubated by goat anti-rabbit (*Gpm6b*, *DR6*) secondary antibody conjugated by horseradish peroxidase (sc-2033, 1 : 3000, Santa Cruz Biotechnology) for 1 h and then washed again by PBS-Tween. Finally, the bands on the membranes were visualized with a SuperSignal West Pico Chemiluminescence Substrate (ECL system from Pierce, #34080) and detected by GBOX visualization system (Syngene). Spot analysis was made using Gene Tools (SynGene).

### 2.5. Data Analysis

All experimental groups consisted of *n* = 110 people. In order to minimize the impact of variability in the experimental people, all samples were measured four times. For the statistical evaluation one-way ANOVA Student-Newmann-Keuls test was used. Data are presented as mean percent ± SD. Statistical analysis was processed by the program GraphPad INSTAT.

## 3. Results and Discussion

The results of molecular detection of *DR6* and *Gpm6B* expression in patients with breast cancer were compared to control group with no tumour predisposition (*n* = 35). We also analysed 75 patients with breast carcinoma.

In the first step of our work we were primarily analysing levels of serum markers like progesterone receptor clone PgR636, p53 clone D07, Ki67 clone MIB-1, and estrogenic receptor clone 1D5 in the experimental group of patients suffering from histopositive breast carcinoma (*n* = 75). Analysis was not performed in eight patients with invasive lobular breast carcinoma. We detected correlation between increased levels of Ki67 clone MIB-1 and increased level of p53 protein clone D07 during grades progression from GI to GIII of breast carcinoma.

Both markers are related to worse prognosis and increased metastatic processes. Although the marker Ki-67 is not determined routinely in present, we found that its levels in G1 stage were by 7.5% higher, in G2 stage were by 19% higher, and in G3 stage were by 54.9% higher than controls. Opposite to this we have found correlation between decreased levels of progesterone and estrogenic receptors. Complete results are shown in Table 2.

**Figure 1:**
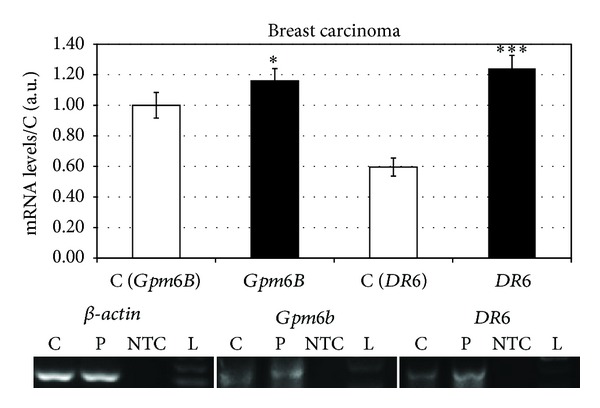
Levels of mRNA of *DR6* and *Gpm6B* in breast carcinoma patients.

**Figure 2:**
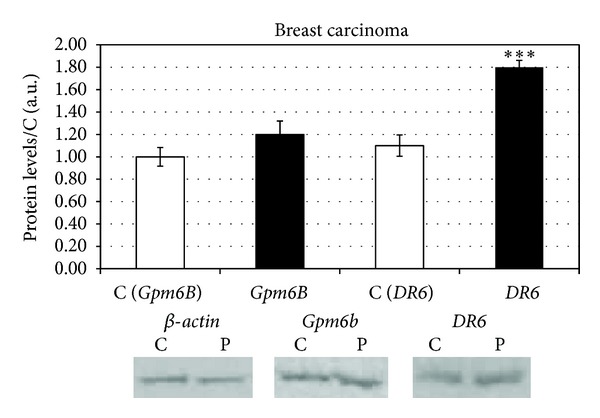
Protein levels of *DR6* and *Gpm6B* in breast carcinoma patients.

In the next step of our work we analysed the mRNA expression of *DR6*, *Gpm6B,* and **β*-actin* genes in peripheral blood of patients (*n* = 75) with breast carcinoma (ductal invasive carcinoma *n* = 65, lobular invasive carcinoma *n* = 8, and nondifferentiated beast carcinoma *n* = 2). In the group of all patients with ductal invasive carcinoma within all grades, we detected, in comparison to control group, significantly increased (106% ± 8.3%) level of *DR6* mRNA (Figure 1), that according to Western blot analysis corresponded with increased level (70% ± 8%) of protein *Dr6* (Figure 2).

During the detection of *Gpm6B* in the same group of patients against control group, we found significantly increased (18 ± 3.1%) levels of *Gpm6B* mRNA (Figure 1). This mRNA level was just partially expressed into slightly higher (20 ± 5.4%) level of protein *Gpm6B*.

**Figure 3:**
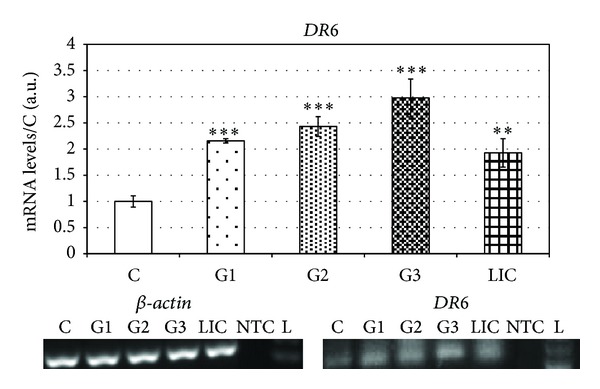
Levels of mRNA of *DR6* in breast carcinoma patients sorted according to grades.

**Figure 4:**
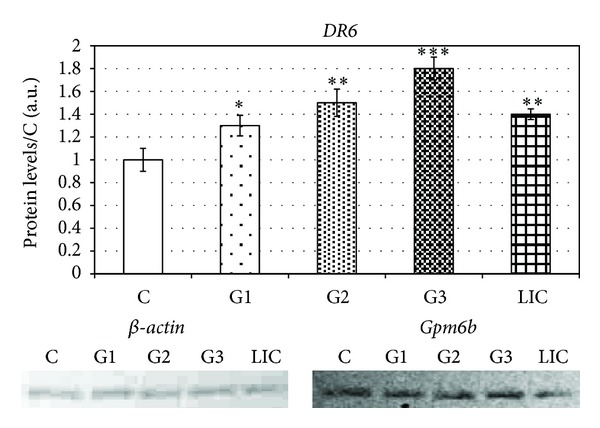
Protein levels of *DR6* in breast carcinoma patients sorted according to grades.

The last stage of our research was aimed at finding the correlation between grade of ductal invasive carcinoma (G1 to G3) and expression of *DR6* and *Gpm6B*. In statistical analysis of *DR6* mRNA level changes we detected significant increase starting in G1 (120 ± 3.2%) and reached maximal level in G3 (197 ± 6.5%) (Figure 3). This expression on mRNA levels was similar to protein levels, which copy rising tendency of mRNA with maximal value in G3 (80 ± 5%) (Figure 4).

**Figure 5:**
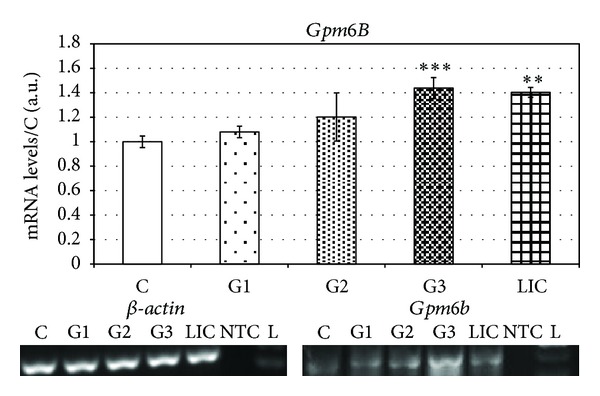
Levels of mRNA of *Gpm6B* in breast carcinoma patients sorted according to grades.

**Figure 6:**
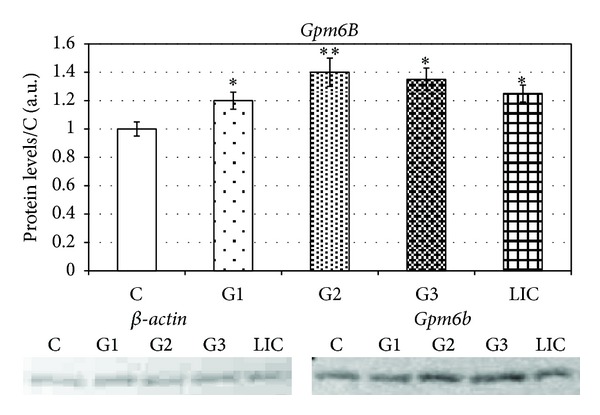
Protein levels of *Gpm6B* in breast carcinoma patients sorted according to grades.

The results of *Gpm6B* were significantly lower. We detected maximal level of mRNA in G3 (40 ± 4.2%) (Figure 5) which corresponded with elevated level of protein *Gpm6B* in the group of patients with the same grade of tumours (35 ± 8.2%) (Figure 6).

We detected significantly increased (92% ± 5.5%) level of *DR6* mRNA in patients suffering from invasive lobular carcinoma (Figure 3). The results correlate with protein levels of *DR6*, where we detected significant increase (40% ± 6%) (Figure 4).

In the levels of *Gpm6B* mRNA from the blood of patients suffering from invasive lobular carcinoma (LIC) we determined increased levels against control group (40 ± 5.3%) (Figure 5).

The levels of protein *Gpm6B* were also significantly increased (22% ± 4.2%) against control group (Figure 6).

All results of mRNA and protein detection are summarized in Table 3.

Neoangiogenesis is a complicated process, regulated by numerous factors simultaneously and in a coordinated fashion. The process of angiogenesis requires that endothelial cells (ECs) detach from pericytes and the extracellular matrix (ECM), proliferate, migrate, and form capillary tubes which connect to other newly developed vascular tubes. A large number of stimulatory angiogenic factors have been discovered including vascular endothelial growth factor (VEGF), basic fibroblast growth factor (b-FGF), interleukin-8, platelet-derived endothelial cell growth factor, tumour necrosis factor alpha, hepatocyte growth factor, angiogenin, transforming growth factor alpha, and transforming growth factor beta [16]. This finding suggests that VEGF functions as a survival factor for tumour vessels. VEGF may prevent EC apoptosis through various mediators including Bcl-2, A1, inhibition of apoptosis (IAP), and the PI3-kinase/Akt [17], MAPK/Erk and SAPK/JNK 9 [16], and signal transduction pathways. Gerber et al. [17] observed upregulation of the antiapoptotic protein Bcl-2 and A1 in VEGF treated ECs. Others have shown that the antiapoptotic effects of VEGF in ECs are mediated by activating MAPK/Erk and inhibiting SAPK/JNK signalling pathways [18].

We were monitoring an expression level of proapoptotic *DR6* and antiapoptotic *Gpm6B* isolated from peripheral blood of patients with increased progression of breast cancer, in both mRNA and protein levels. The death ligands and receptors of the TNF/TNFR family have emerged as attractive candidates for modulating tumour cell apoptosis based on their ability to bypass p53-dependent mechanisms in executing programmed cell death [19]. In some cells, called type 1, the DR-initiated extrinsic pathway generates a signal strong enough to initiate apoptosis by itself. However, in the majority of cells, called type 2, the DR-initiated signal needs to be amplified to induce apoptosis. This amplification can be achieved by a cross-talk between the extrinsic pathway and the Bcl-2 regulated mitochondrial, or intrinsic, pathway [20]. One mechanism by which the extrinsic pathway recruits the intrinsic pathway involves caspase-8 mediated cleavage of the proapoptotic BH3-only Bcl-2 family member, Bid, to generate active truncated Bid. Active truncated Bid antagonizes the function of prosurvival Bcl-2 family members, such as Bcl-2 and Bcl-XL, triggering a sequence of events that culminates in the release of apoptotic factors from the mitochondrion [21]. These apoptotic factors mediate activation of caspase-9 and antagonize the activity of inhibitors of apoptosis that otherwise function to suppress caspase activity.

Kasof et al. [22] report that the new member of the TNFR family, *DR6*, induced apoptosis. A primary function for death receptors is to induce apoptosis [23]. Additionally, *DR6* may regulate the cytokine-driven differentiation of monocytes to dendritic cells, which suggests, *DR6* could play a role in the development of myeloid derived suppressor cells within tumours [14]. *DR6* has been reported to be upregulated in numerous solid tumours [20]. *DR6* is expressed ubiquitously with high expression in lymphoid organs, heart, brain, and pancreas. Ectopic expression of *DR6* in some cell lines leads to apoptosis and activation of the JNK and NF-*κ*B pathways. Some tumour cells overexpress *DR6*, typically in conjunction with elevated antiapoptosis molecules [14]. *DR6* is expressed not only in cancer cells, but also in tumour vascular cells. This expression on host cells in the tumour microenvironment suggests that *DR6* may have broad applicability as a tumour biomarker. Recently it was shown that soluble *DR6* is the result of matrix metalloproteinase-14 (MMP-14) cleavage of membrane bound *DR6* [24]. Thus serum *DR6* protein may serve as an indicator of tumour MMP-14 levels. MMP-14 has been correlated with tumour invasion, metastases, and poor patient prognosis [25]. We examined significantly increased mRNA levels of *DR6* in all three experimental groups (GI–GIII). The mRNA levels of *DR6* in patients suffering from breast carcinoma were by 106% higher than in control group. The mRNA levels correlated with the levels of particular proteins. We observed significantly increased (70%) level of *DR6* protein in breast carcinoma. Protein levels were increased in G1 by 30%, in G2 by 50%, and in G3 by 80% (Table 3).

These findings correspond with results of Buckanovich et al. [5], who pointed out the increase of *DR6* mRNA level by 152% comparing to healthy patients. Our results as well correlate with Yang et al. [26] who detected overexpression (125%) of *DR6* mRNA in patients suffering from ductal invasive carcinoma.

We were also monitoring the changes in expression of antiapoptotic *Gpm6B*. Discovery of relationship between coexpressions of SERT and *Gpm6B* which mediates a significant decrease in SERT-mediated 5-HT-uptake is very important proof of antiapoptotic activity of *Gpm6B* [10]. 5-HT does not enhance tumour cell proliferation but acts as a regulator of angiogenesis by reducing the expression of matrix metalloproteinase-12 (MMP-12) in tumour-infiltrating macrophages. Tumour-infiltrating macrophages are associated with tumour progression and metastasis. They were reported to regulate angiogenesis in breast (Lin et al.) [27] as well as lung cancer and melanoma (Houghton et al.) [28]. Macrophages are known to specifically secrete MMP-12, which leads to enhanced generation of angiostatin [29] and thereby suppresses tumour growth by halting angiogenesis. We found elevated levels of *Gpm6B* mRNA in all grades of breast cancer with maximal level in G3. In protein levels we detected maximal level in GII with the constant level of expression in G3. These results showed that antiapoptotic signalling during neovascularization is significantly increased. Building on the results, it will be advisable in the future on study the influence of cytostatic treatment to the expression of genes related to apoptotic pathways and their relationship with progression of breast cancer tumours.

## 4. Conclusion

Formation of new blood vessels and metastasis are two processes that are central to the progression of cancer. As such, they have become important targets for the development of anticancer agents. The paper describes the expression of *Dr6* and *Gpm6B* as members of apoptotic machinery in correlation with intensity of neovascularization during tumour growth and metastatic processes. We found significant changes of mRNA expression levels in tumour vascular genes *DR6* and *Gpm6B* obtained from peripheral blood of patients suffering from different grades of breast carcinoma. The study also refers to increase of tumour vascular proteins levels coded by *DR6* and *Gpm6B* genes. After confrontation of the results obtained by TVM clinical, biochemical, and histological detection, we found out that increased levels of *DR6* proteins in G1 to G3 stages are similar to increased Ki-67 clone MIB-1. In comparison between *DR6* and *Gpm6B* protein levels it was obvious that protein levels of *DR6* were about 60% higher than *Gpm6B* which suggests higher proapoptotic signalization mediated through TNF/TNFR family members.

The study of expression of apoptotic machinery members during neovascularization provides an explanation of the role of angiogenic factors in correlation with breast cancer progression and suggests that this approach could help with the development of effective antiangiogenic therapies useful for treatment of breast cancer.

## Figures and Tables

**Table 1:** Primer sequences and length of amplified fragments.

Primers/forward/reverse/antibodies	Sequence	Fragment length (bp)	Distributor
*β*-actin/forward/	ACACAGGGGAGGTGATAGCAT	110 bp	Invitrogen
*β*-actin/reverse/	ATACATCTCAAGTTGGGGGACAA	110 bp	Invitrogen
*DR6*/forward/	CCGCCGAGCCACAGCCACGAT	384 bp	Invitrogen
*DR6*/reverse/	CCCTTTCTTCCGCACACCCCAACC	384 bp	Invitrogen
*Gpm6B*/forward/	TCCTATCACCTGTTCATTGTGG	171 bp	Invitrogen
*Gpm6B*/reverse/	GCAGCAATCTTCCCGACTC	171 bp	Invitrogen

**Table 2:** Results of molecular analysis of breast carcinoma using histochemical detection.

Analytical stage	ER > 1% clon 1D5	PR > 1% clon PgR636	Ki67 % clon MIB-1	p53 clon D 07
G 0	64	46.4	11.2	33.15
G I	95	86.25	7.5	41.5
G II	81	68	19	12
G III	32,5	29.16	54.16	73.3

**Table 3:** Correlation between grade of breast carcinoma and expression of *DR6* and *Gpm6B*.

Grade of breast cancer against controls	*DR6 *		*Gpm6B *	
	mRNA levels of *DR6* (%)	Protein *DR6* (%)	mRNA levels of *Gpm6B* (%)	Protein *Gpm6B* (%)
Breast carcinoma average values	↑106	↑70	↑16	↑20
G I	↑110	↑30	↑6	↑20
G II	↑143	↑50	↑20	↑40
G III	↑197	↑80	↑40	↑30

## Abbreviations

DRS: Death receptors systems
EC: Endothelial cell
G1: Grade1
G2: Grade2
G3: Grade3
*Gpm6B*: Glycoprotein M6-b
LIC: Lobular invasive carcinoma
NF-kB: Nuclear factor kappa-light-chain-enhancer of activated B cells
NF-UB: Ubiquitin signalling in the nf-kappab pathway
PgR636: Progesterone receptor
PLP: Proteolipid protein
SDS-PAGE: Sodium dodecyl sulphate-polyacrylamide gel electrophoresis
TNF: Tumour necrosis factor group
TNFR1: Tumour necrosis factor receptor
TRAIL: TNF-related apoptosis-inducing ligand
TVM: Tumour vascular markers
VEGF: Vascular endothelial growth factor
VLC: Vascular leukocytes.
